# Transcriptome analysis of differentially expressed unigenes involved in flavonoid biosynthesis during flower development of *Chrysanthemum morifolium* ‘Chuju’

**DOI:** 10.1038/s41598-018-31831-6

**Published:** 2018-09-07

**Authors:** Junyang Yue, Chuanxue Zhu, Yu Zhou, Xiangli Niu, Min Miao, Xiaofeng Tang, Fadi Chen, Weiping Zhao, Yongsheng Liu

**Affiliations:** 1 School of Food Science and Engineering, Hefei University of Technology, Hefei, 230009 China; 2 School of Computer and Information, Hefei University of Technology, Hefei, 230009 China; 3 School of Horticulture, Anhui Agricultural University, Hefei, 230036 China; 4 College of Horticulture, Key Laboratory of Landscape Agriculture, Ministry of Agriculture, Nanjing Agricultural University, Nanjing, 210095 China; 5 School of Biological Science and Food Engineering, Chuzhou University, Chuzhou, 239000 China

## Abstract

*Chrysanthemum morifolium* is an ornamentally and medicinally important plant species. Up to date, molecular and genetic investigations have largely focused on determination of flowering time in the ornamental species. However, little is known about gene regulatory networks for the biosynthesis of flavonoids in the medicinal species. In the current study, we employed the high-throughput sequencing technology to profile the genome-wide transcriptome of *C. morifolium* ‘Chuju’, a famous medicinal species in traditional Chinese medicine. A total of 63,854 unigenes with an average length of 741 bp were obtained. Bioinformatic analysis has identified a great number of structural and regulatory unigenes potentially participating in the flavonoid biosynthetic pathway. According to the comparison of digital gene expression, 8,370 (3,026 up-regulated and 5,344 down-regulated), 1,348 (717 up-regulated and 631 down-regulated) and 944 (206 up-regulated and 738 down-regulated) differentially expressed unigenes (DEUs) were detected in the early, middle and mature growth phases, respectively. Among them, many DEUs were implicated in controlling the biosynthesis and composition of flavonoids from the budding to full blooming stages during flower development. Furthermore, the expression patterns of 12 unigenes involved in flavonoid biosynthesis were generally validated by using quantitative real time PCR. These findings could shed light on the molecular basis of flavonoid biosynthesis in *C. morifolium* ‘Chuju’ and provide a genetic resource for breeding varieties with improved nutritional quality.

## Introduction

*Chrysanthemum morifolium*, a species of herbaceous perennial plant from Asteraceae family, is commonly called Flos chrysanthemi in Latin and Juhua in mandarin^1^. It is native of China although nowadays is widely cultivated worldwide. As one of the most important ornamental species, *C. morifolium* has already become the second economically most important floricultural crop following rose and its industry is still flourishing due to a steadily increased demand in cut flower market^2^. Furthermore, the dry capitulum of *C. morifolium* is also a medicinal and edible cognate that has been used as an herbal tea or a food supplement for over 2000 years of history in China^3^.

Through a long time of selection, many commercial cultivars of *C. morifolium* are used specially for their curative effects rather than esthetic values^4^. According to growing regions, the main medicinal cultivars available in the herb markets are divided into Boju (Bozhou, Anhui Province), Chuanju (Zhongjiang, Sichuan Province), Chuju (Chuzhou, Anhui Province), Hangju (Tongxiang, Zhejiang Province), Huaiju (Wuzhi, Henan Province), Qiju (Anguo, Hebei Province), Yanju (Yancheng, Jiangsu Province). Among them, Chuju, Hangju, Huaiju and Boju are the four cultivars with highest reputations due to their quality and application in traditional Chinese medicine (TCM)^4^. Accumulating pharmacological studies have shown that the capitulum of medicinal *C. morifolium* possesses significant amounts of natural antioxidants, which execute essential functions in anti-inflammatory, anti-bacterial, anti-tumor and cardiovascular protective^5–8^. Therefore, it is important to better understand molecular mechanisms involved in the biosynthesis and accumulation of antioxidant metabolites throughout the flower development of *C. morifolium*.

Flavonoids, a class of powerful antioxidant compounds, are very rich and primarily responsible for the aforementioned bioactivities in medicinal *C. morifolium*^9^. They are synthesized by starting with the phenylpropanoid pathway and subsequently leading to nine different metabolic branches, including the products of chalcones, aurones, isoflavonoids, flavones, flavonols, flavandiols, anthocyanins, condensed tannins, and phlobaphene^10^. Up to now, flavonoid-related genes have been extensively characterized through both forward and reverse genetic approaches in a range of model species^11^. But in *C. morifolium*, only several homologous genes, such as flavonoid 3′-hydroxylase (F3′H), flavonoid 3′,5′-hydroxylase (F3′5′H) and cytochrome P450 have been isolated so far^12–14^. The majority of genes that involve in the flavonoid accumulation are not yet known in the medicinal *C. morifolium* species.

Total RNA sequencing (RNA-Seq) could provide comprehensive insights into biological pathways by revealing the presence and quantity of gene expression levels^15^. In *C. morifolium* ‘Fenditan’, a large number of transcription factors and structural genes involved in the photoperiod pathway as well as flower organ determination have been identified from whole transcriptome data by using the RNA-Seq technology^16^. Meanwhile, a transcriptome analysis of another cultivar *C. morifolium* ‘Yuuka’ succeeded in identifying many differentially expressed genes under the treatment of various daylengths^17^. While this technology has been intensively applied for studying flowering-time regulation in ornamental *C. morifolium* cultivars, but to our knowledge, no transcriptomic analysis regarding the flavonoid biosynthesis has been reported in medicinal *C. morifolium* cultivars.

In the present study, whole transcriptome sequencing and comprehensive comparative analysis were performed using flowers of four sequentially developmental stages to identify flavonoid-associated genes in *C. morifolium* ‘Chuju’, a cultivar derived from TCM with top medicinal quality. Bioinformatics analysis of the sequencing data revealed a wide range of unigenes putatively participating in the flavonoid pathway, some of which represented differential expression patterns across the whole period of flower development. These results we obtained could help further elucidating the molecular mechanisms of flavonoid biosynthesis and better understanding the regulatory networks of gene expression.

## Results

### Transcriptome sequencing and *de novo* assembly

**Figure 1:**
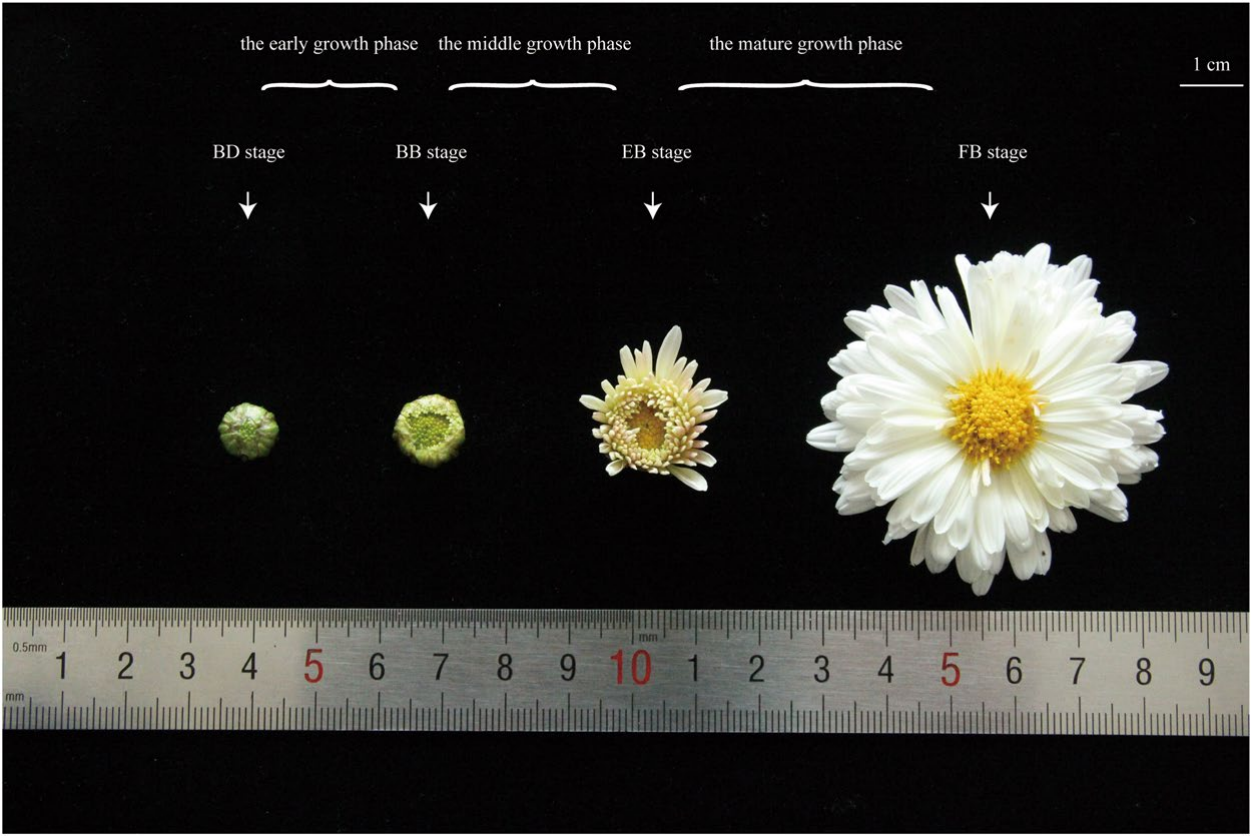
The four selected stages of flower development in *C. morifolium* ‘Chuju’: budding stage (BD stage), bud breaking stage (BB stage), early blooming stage (EB stage), and full blooming stage (FB stage). The stages between BD and BB were denoted as the early growth phase. The stages between BB and EB were denoted as the middle growth phase. The stages between EB and FB were denoted as the mature growth phase. Specially, the scale bar in the top right represents 1 cm in length.

To obtain a comprehensive view of candidate genes involved in the flavonoid biosynthetic pathway, we have pooled and sequenced equivalent quantities of RNA extracts isolated from four sequential stages throughout flower development in *C. morifolium* ‘Chuju’, which were denoted as the budding (BD), bud breaking (BB), early blooming (EB) and full blooming (FB) stages (Fig. 1, detailed in Methods). In total, 46,527,128 raw reads were generated using the RNA-Seq technology. After removing adaptors, cleaning up contaminations and filtering out low quality reads, 38,454,857 clean reads with a total data of 7,767,261,963 bp (7.77 GB) were obtained. Their average Q30 and GC percentage were 95.07% and 43.16%, respectively. Based on the high-quality data, 3,475,234 contigs with 225,683,083 bp of data were subsequently assembled. Finally, 195,160 transcripts and 63,854 unigenes were successively recognized from these contigs via the paired-end relationships. The non-redundant unigenes obtained were associated with an average length of 741 bp and an N50 value of 1,234 bp (Table 1). The raw sequencing data have been deposited at the Sequence Read Archive (SRA) of the National Center for Biotechnology Information (NCBI) database under accession number PRJNA397042 (http://www.ncbi.nlm.nih.gov/bioproject/PRJNA397042).

**Table 1:** Summary for the transcriptome assembly.

Assembly	Total number	Total length	N50 length	Average length
Contig	3,475,234	225,683,083	65	64.94
Transcript	195,160	174,260,788	1,282	892.91
Unigene	63,854	47,320,610	1,234	741.08

### Gene annotation and functional classification

**Figure 2:**
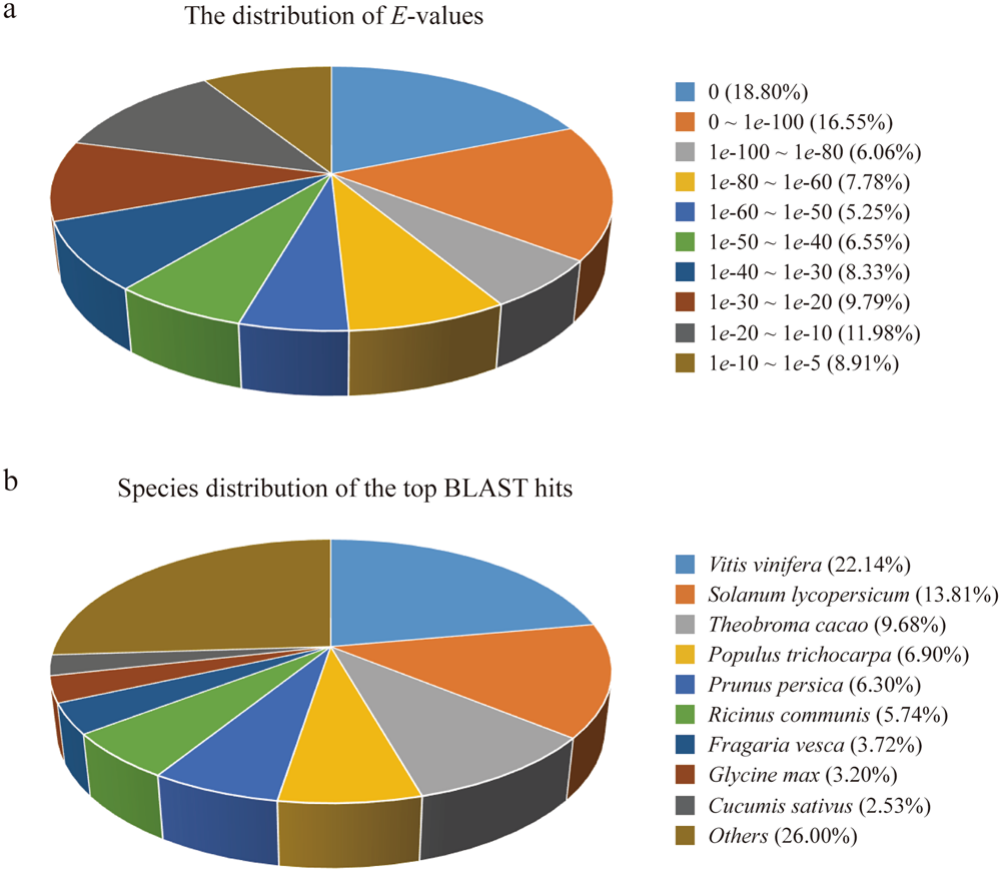
(**a**) Statistical analysis of the distributed *E*-value from the alignment with available plant sequences in the nr database. (**b**) Species distribution of the top BLAST hits for the best alignment against the nr database.

In order to obtain annotations of the assembled unigenes, their sequences were aligned against a series of publicly available nucleotide and protein databases. Of all these 63,854 unigenes, 34,362, 23,579, 7,694 and 10,578 were respectively aligned to the NCBI non-redundant (nr), Swiss-Prot, Kyoto encyclopedia of genes and genomes (KEGG) and Clusters of orthologous groups (COG) databases by using an *E*-value cutoff of 1*e*−5 (Table 2). After eliminating redundancy from different databases, a total of 34,605 unigenes were annotated at least once, accounting for approximately 54.19%. Among them, 34,362 could be annotated to the nr database, covering up to 99.30% of the total annotated unigenes. A statistical analysis of the distributed *E*-value has revealed that 79.02% of the mapped sequences have strong homologies (*E*-value < 1*e*-20) and 54.44% sequences have extremely strong homologies (*E*-value < 1*e*-50) to the available plant sequences in the nr database (Fig. 2a). Furthermore, species distribution of the top BLAST hits for the best alignment against the nr database was presented in Fig. 2b. The top scoring is *Vitis vinifera* (22.14%), followed by *Solanum lycopersicum* (13.81%) and *Theobroma cacao* (9.68%). In comparison, there are only 3.02% of the aligned sequences from the Asteraceae species, including *Artemisia* (0.52%), *Carthamus* (0.07%), *Chrysanthemum* (0.40%), *Gerbera* (0.11%), *Helianthus* (1.26%), *Lactuca* (0.18%), *Senecio* (0.07%) and *Zinnia* (0.25%).

**Table 2:** Annotation of unigenes against diverse databases.

Public database	Count	%
nr	34,362	53.81
Swiss-Prot	23,579	36.93
KEGG	7,694	12.05
COG	10,578	16.57
GO	25,394	39.77
Total	34,605	54.19

**Figure 3:**
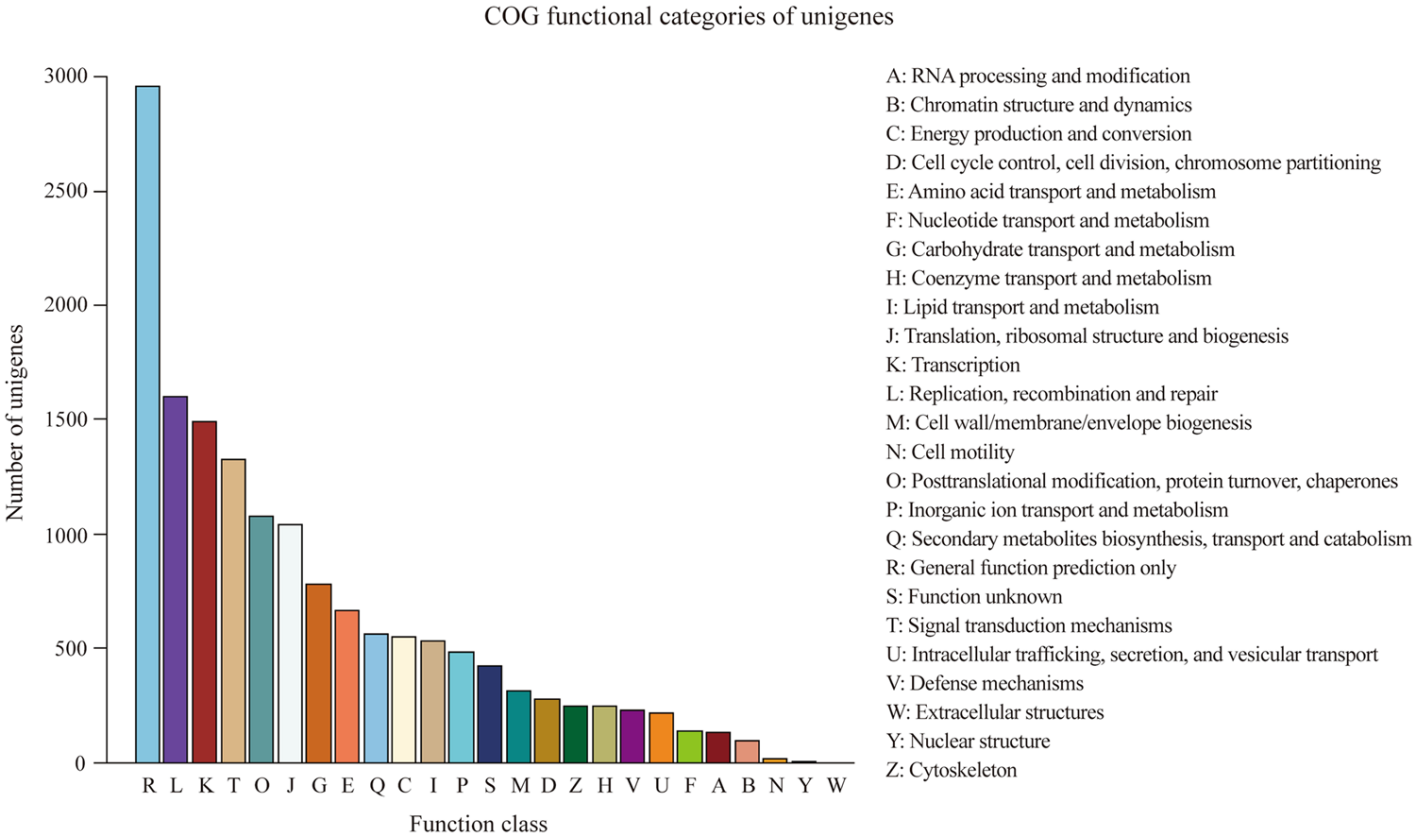
A total of 10,578 unigenes were distributed in 25 different COG functional categories in *C. morifolium* ‘Chuju’.

The Swiss-Prot database was reviewed by manual curation, consequently representing a high quality and accuracy. In the present study, 23,579 unigenes were annotated to the Swiss-Prot database, with a sharing of 23,386 annotated unigenes to the nr database. In addition, 7,694 and 10,578 unigenes were mapped into 118 KEGG pathways and 25 COG categories, respectively. The KEGG pathways for ‘metabolic pathways’ represented the largest group, followed by ‘biosynthesis of secondary metabolites’, ‘ribosome’, ‘protein processing in endoplasmic reticulum’, ‘plant hormone signal transduction’, ‘spliceosome’ and ‘RNA transport’ (Supplementary Table S1). Among the 25 COG categories, the clusters that frequently mapped were ‘general function prediction only’, ‘replication, recombination and repair’, ‘transcription’, ‘signal transduction mechanisms’ and ‘posttranslational modification, protein turnover, chaperones’ (Fig. 3).

**Figure 4:**
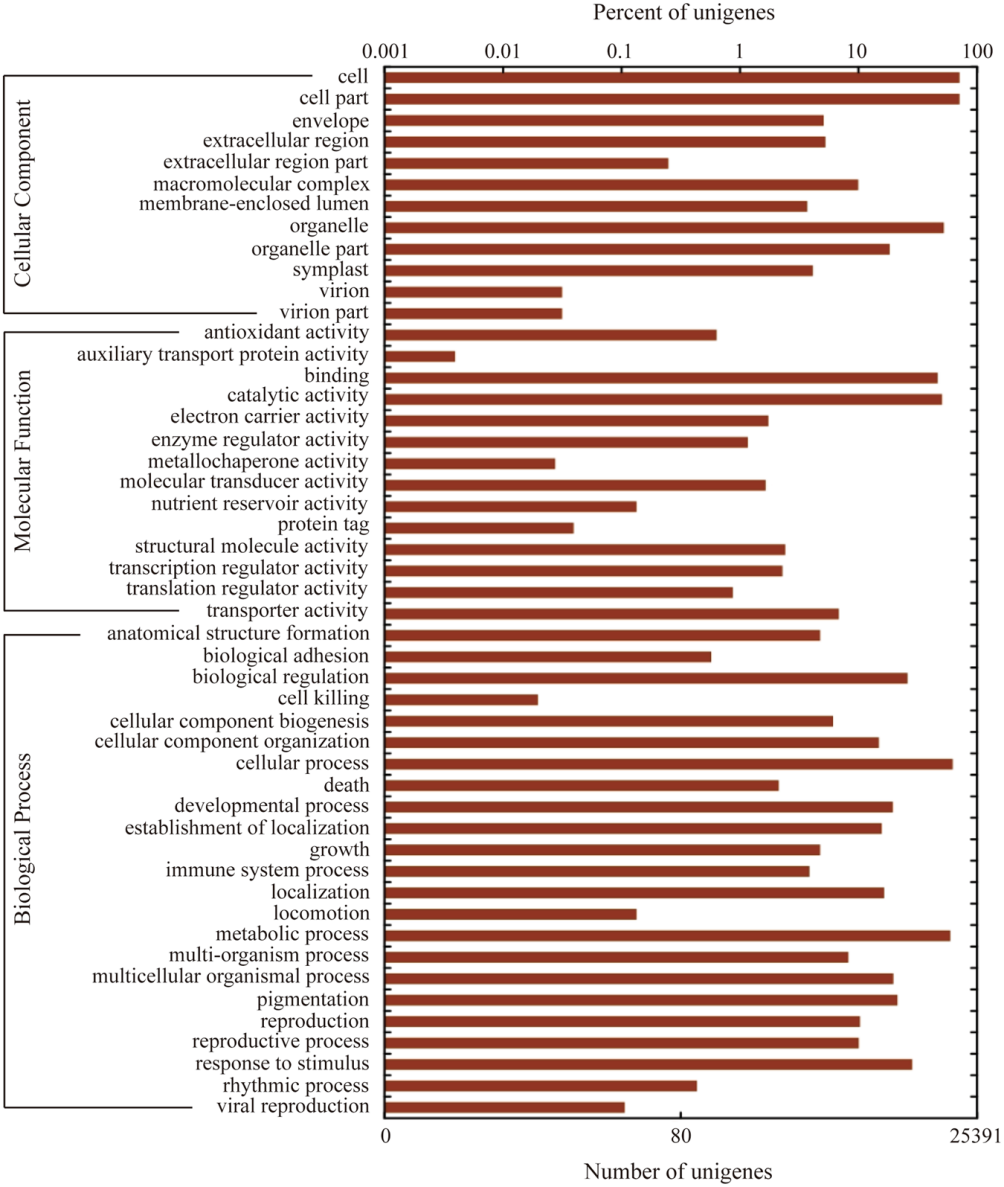
GO classification of the unigenes were summarized (in level 2) in the three main categories: biological process, molecular function and cellular component.

On the basis of Gene Ontology (GO) annotation, 25,394 unigenes could be assigned to at least one GO category using the Blast2GO pipeline^18^. Among them, 19,856 unigenes were classified into the biological process category, 18,447 unigenes were classified into the cellular component category, and 19,130 unigenes were classified into the molecular function category. As summarized at the level 2, a total of 49 functional GO terms were annotated (Fig. 4). For each of the three main categories, the dominant GO terms were ‘cellular process’ (in ‘biological process’), ‘cell or cell part’ (in ‘cellular component’) and ‘catalytic activity’ (in ‘molecular function’), which indicated that the basic cellular process in cells were catalytic activities during flower development of *C. morifolium* ‘Chuju’. Also, the high percent of ‘metabolic process’ (in ‘biological process’) and ‘binding’ (in ‘molecular function’) might suggest that metabolic processes are regulated by a wide range of functional protein complex through interacting with each other. By contrast, there were relatively few genes from ‘locomotion’ (in ‘biological process’), ‘extracellular region part’ (in ‘cellular component’) and ‘translation regulator activity’ (in ‘molecular function’) in the three main categories, respectively.

### Digital gene expression profiling and *in silico* analysis

**Figure 5:**
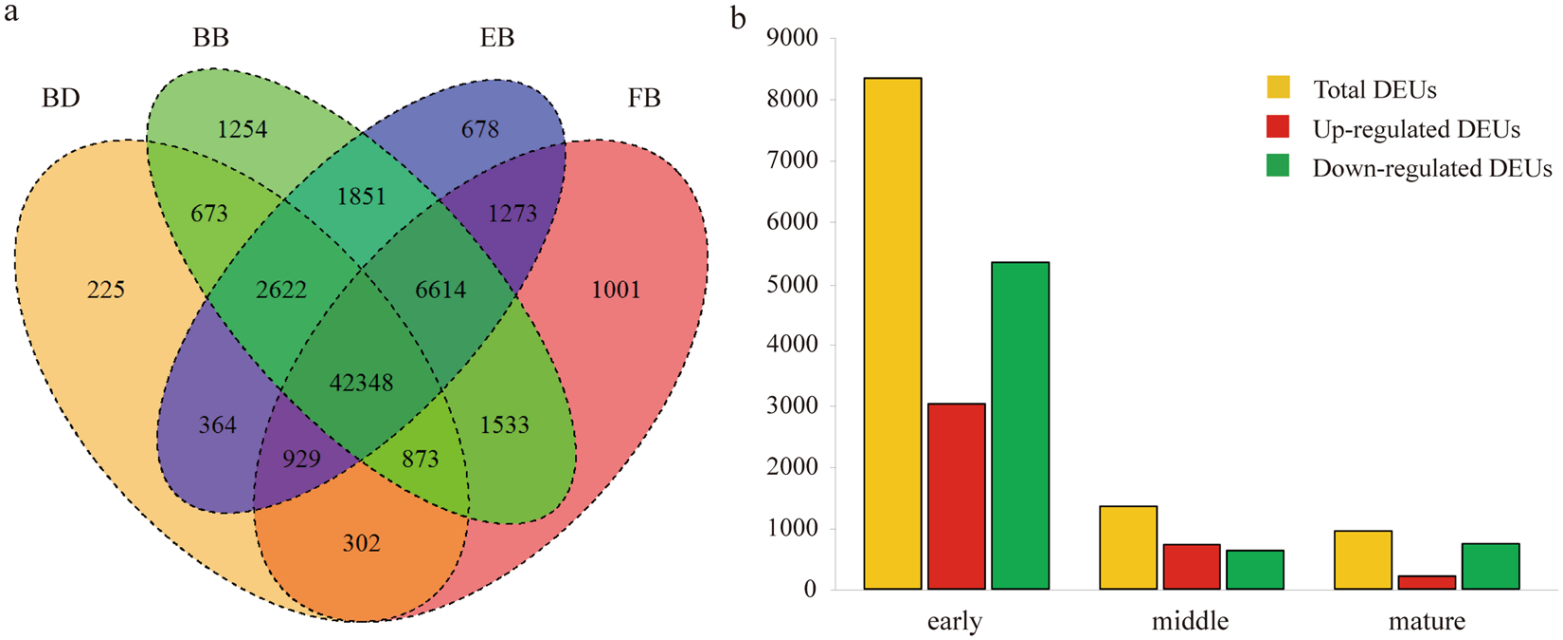
(**a**) Venn diagram of the number of unigenes with a RPKM value of >0.1 in the budding (BD), bud breaking (BB), early blooming (EB) and full blooming (FB) stages. (**b**) The differentially expressed unigenes (DEUs) were identified in the early (BD-BB), middle (BB-EB) and mature (EB-FB) growth phases.

To explore the patterns of gene expression throughout flower development, digital gene expression (DGE) profiling was also performed for each flower sample from the BD, BB, EB and FB stages of *C. morifolium* ‘Chuju’ by using the RNA-Seq technology. As a result, the average number of raw reads generated from these four samples was 10,432,456, 10,324,977, 11,330,110 and 10,814,843, respectively. After filtering and cleaning, 7,560,601, 8,270,676, 8,813,243 and 8,561,427 high-quality reads were obtained on average. Using the assembled unigenes as references, 48,336, 57,768, 56,679 and 54,873 sequences were respectively detected with a threshold RPKM (reads per kilobase per million mapped reads) value of 0.1. Among them, 42,348 unigenes were commonly expressed in all the four developmental stages, accounting for most of the identified unigenes. On the other hand, only 225, 1,254, 678 and 1,001 unigenes were specifically expressed in the BD, BB, EB and FB stages, respectively (Fig. 5a). These expression profile data were also deposited at the SRA database under the accession number PRJNA397042 (http://www.ncbi.nlm.nih.gov/bioproject/PRJNA397042).

Subsequently, differentially expressed unigenes (DEUs) were identified between every two sequential stages, including the early (between the BD and BB stages), middle (between the BB and EB stages) and mature (between the EB and FB stages) growth phases. The comparative results revealed that there were 8,370 DEUs (3,026 up-regulated and 5,344 down-regulated) in the early growth phase, 1,348 DEUs (717 up-regulated and 631 down-regulated) in the middle growth phase, and 944 DEUs (206 up-regulated and 738 down-regulated) in the mature growth phase (Fig. 5b). As shown in the figure, an overall decreasing number of unigenes were differentially expressed during flower development of *C. morifolium*.

**Figure 6:**
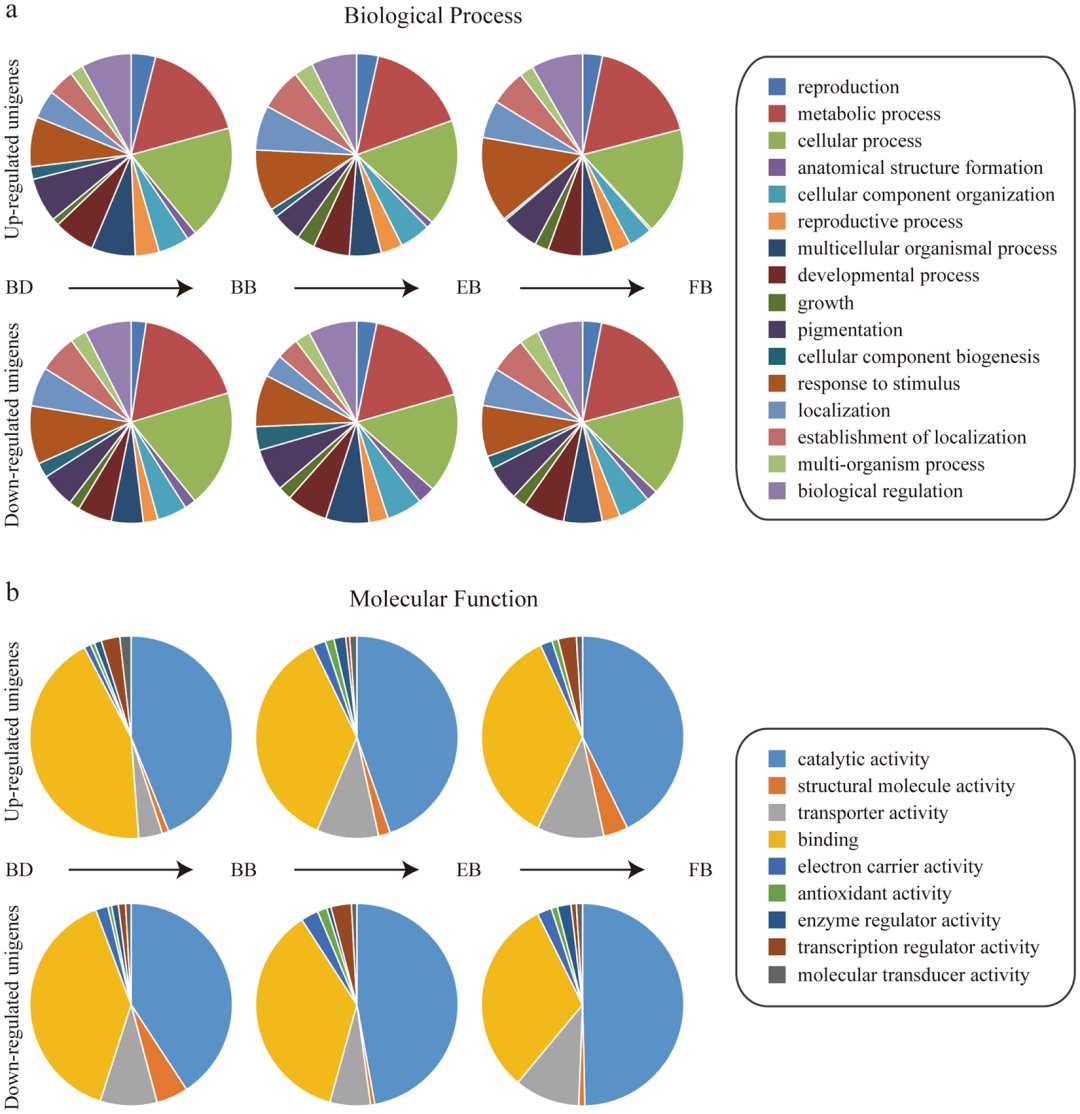
GO annotation of differentially expressed unigenes (DEUs) in the early (BD-BB), middle (BB-EB) and mature (EB-FB) growth phases. (**a**) The biological process category. (**b**) The molecular function category.

In order to inspect the functional classification of these DEUs, all the unigenes involved were assigned to biological process and molecular function categories according to the GO annotation^19^. Generally, a great number of functional enzymes and regulatory proteins were identified (Fig. 6). Using the entire unigenes assembled from transcriptome analysis as background, significantly enriched GO terms for DEUs in each growth phase were obtained based on the Hypergometric test. In the early growth phase, the up-regulated unigenes were significantly enriched in ‘microtubule binding’ (in ‘molecular function’), ‘microtubule motor activity’ (in ‘molecular function’) and ‘regulation of flower development’ (in ‘biological process’) (Supplementary Table S2), while the down-regulated unigenes were significantly enriched in ‘chlorophyll binding’ (in ‘molecular function’), ‘response to cadmium ion’ (in ‘biological process’) and ‘water transport’ (in ‘biological process’) (Supplementary Table S3). In the middle growth phase, the up-regulated unigenes were significantly enriched in ‘plant-type cell wall modification’ (in ‘biological process’), ‘pollen tube growth’ (in ‘biological process’) and ‘hydrogen-exporting ATPase activity, phosphorylative mechanism’ (in ‘molecular function’) (Supplementary Table S4), while the down-regulated unigenes were significantly enriched in ‘cell proliferation’ (in ‘biological process’), ‘nucleosome assembly’ (in ‘biological process’) and ‘DNA unwinding involved in DNA replication’ (in ‘biological process’) (Supplementary Table S5). In the mature growth phase, the up-regulated unigenes were significantly enriched in ‘regulation of cellular component organization’ (in ‘biological process’), ‘protein complex subunit organization’ (in ‘biological process’) and ‘phosphatidylinositol phosphate binding’ (in ‘molecular function’) (Supplementary Table S6), while the down-regulated unigenes were significantly enriched in ‘fatty acid metabolic process’ (in ‘biological process’), ‘alkane 1-monooxygenase activity’ (in ‘molecular function’) and ‘ATP hydrolysis coupled proton transport’ (in ‘biological process’) (Supplementary Table S7).

### Characterization of functional genes involved in flavonoid biosynthesis

Generally, genes involved in the flavonoid pathway are divided into two categories: structural genes that encode flavonoid biosynthetic enzymes and regulatory genes that control the expression of structural genes^20^. According to existing experimental findings, the central pathway for flavonoid biosynthesis is conserved in plants and many functional genes involved have been extensively characterized in model species^21^. Based on bioinformatics analysis from transcriptome of *C. morifolium*, many unigenes were identified as homologous sequences of the major structural genes (Table 3), including *chalcone synthase* (*CHS*), *chalcone isomerase* (*CHI*), *flavone synthase* (*FNS*), *flavanone 3-hydroxylase* (*F3H*), *flavonoid 3*′*-hydroxylase* (*F3*′*H*), *flavonoid 3*′*,5*′*-hydroxylase* (*F3'5*′*H*), *flavonol synthase* (*FLS*), *dihydroflavonol 4-reductase* (*DFR*), *anthocyanidin synthase* (*ANS*) and *UDP-glucose-flavonoid 3-O-glucosyltransferase* (*3GT*). Meanwhile, 1,077 unigenes were predicted as transcription factors and distributed in 66 different families by using the iTAK online program (Supplementary Table S8)^22^. Among them, the most frequently occurring was the C2H2 family, followed by the AP2/ERF-ERF, bHLH, MYB and MYB-related families. With respect to transcription factors involved in the regulation of flavonoid biosynthesis, the bHLH, MYB and MYB-related families have been extensively documented to activate or repress transcription of specific target genes through formation of the highly dynamic MYB-bHLH-WD40 (MBW) complex in a number of plant species^23,24^. Specially, the common function of WD40 proteins is coordinating multiple proteins to form complex assemblies by protein-protein interactions and a total of 37 homologous members were identified in this study.

**Table 3:** Homologous sequences of the major structural genes involved in flavonoid biosynthesis.

Gene name	Number of unigenes	Unigene ID
*CHS*	14	c101558.graph_c0, c40871.graph_c0, c44663.graph_c0, c44663.graph_c1, c45359.graph_c0, c58833.graph_c0, c66249.graph_c0, c69338.graph_c0, c70330.graph_c0, c71660.graph_c0, c75718.graph_c0, c76491.graph_c0, c83126.graph_c0, c95501.graph_c0
*CHI*	4	c58710.graph_c0, c75201.graph_c0, c82925.graph_c0, c92719.graph_c0
*FNS*	2	c80466.graph_c0, c80721.graph_c0
*F3H*	10	c43327.graph_c0, c56548.graph_c0, c70708.graph_c0, c72593.graph_c0, c72723.graph_c0, c74491.graph_c0, c80223.graph_c0, c82203.graph_c0, c82856.graph_c0, c85193.graph_c0
*F3′H*	7	c65196.graph_c2, c81046.graph_c0, c81361.graph_c0, c82449.graph_c0, c82454.graph_c0, c84510.graph_c0, c84810.graph_c0
*F3′5′H*	18	c1164.graph_c0, c13046.graph_c0, c45583.graph_c0, c49253.graph_c0, c49253.graph_c1, c52937.graph_c0, c58682.graph_c0, c61195.graph_c0, c69911.graph_c3, c70120.graph_c0, c70561.graph_c0, c77991.graph_c0, c78510.graph_c0, c88709.graph_c0, c88745.graph_c0, c98782.graph_c0, c99756.graph_c0, c99797.graph_c0
*FLS*	17	c100176.graph_c0, c33294.graph_c0, c44831.graph_c0, c54600.graph_c0, c54600.graph_c1, c54729.graph_c0, c66274.graph_c0, c66380.graph_c0, c71332.graph_c0, c74830.graph_c0, c74864.graph_c0, c74864.graph_c1, c75782.graph_c0, c77282.graph_c0, c78657.graph_c0, c82790.graph_c0, c92537.graph_c0
*DFR*	8	c42268.graph_c0, c57810.graph_c0, c65632.graph_c0, c66947.graph_c0, c81523.graph_c0, c81533.graph_c0, c81543.graph_c0, c83366.graph_c0
*ANS*	4	c81731.graph_c0, c82899.graph_c0, c85476.graph_c0, c87171.graph_c0
*3GT*	13	c38996.graph_c0, c68500.graph_c0, c68500.graph_c1, c68500.graph_c2, c74006.graph_c0, c82202.graph_c0, c82623.graph_c0, c82766.graph_c0, c83678.graph_c0, c89757.graph_c1, c89757.graph_c2, c89941.graph_c1, c96328.graph_c0

**Figure 7:**
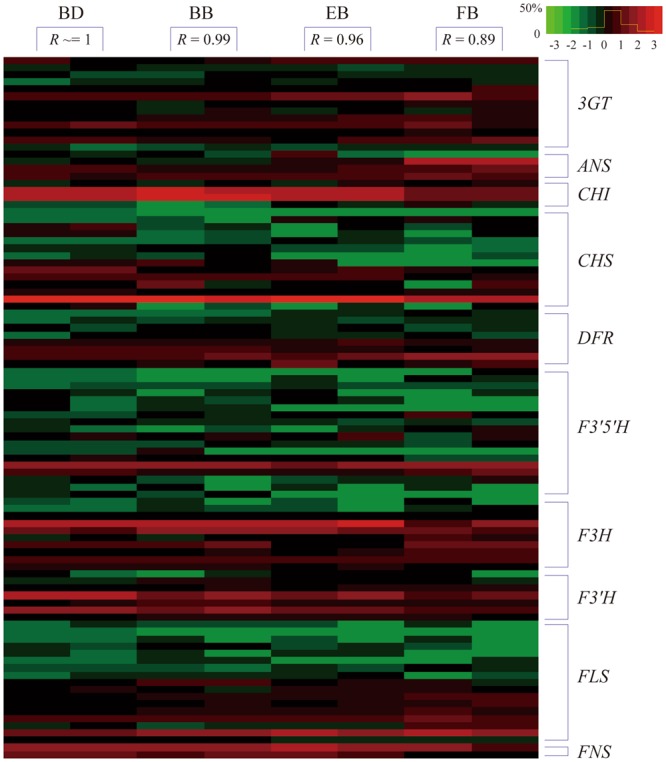
Heatmap of the expression levels of structural unigenes related to flavonoid biosynthesis in the budding (BD), bud breaking (BB), early blooming (EB) and full blooming (FB) stages. Repeated measures were taken for each stage and the pearson correlation coefficient (*R*) is calculated between each replicate. The color scale indicates a relative fold-change in gene expression, where red indicates high expression and green indicates low expression. The yellow line within the color scale indicates the percent of unigenes with different interval values.

**Figure 8:**
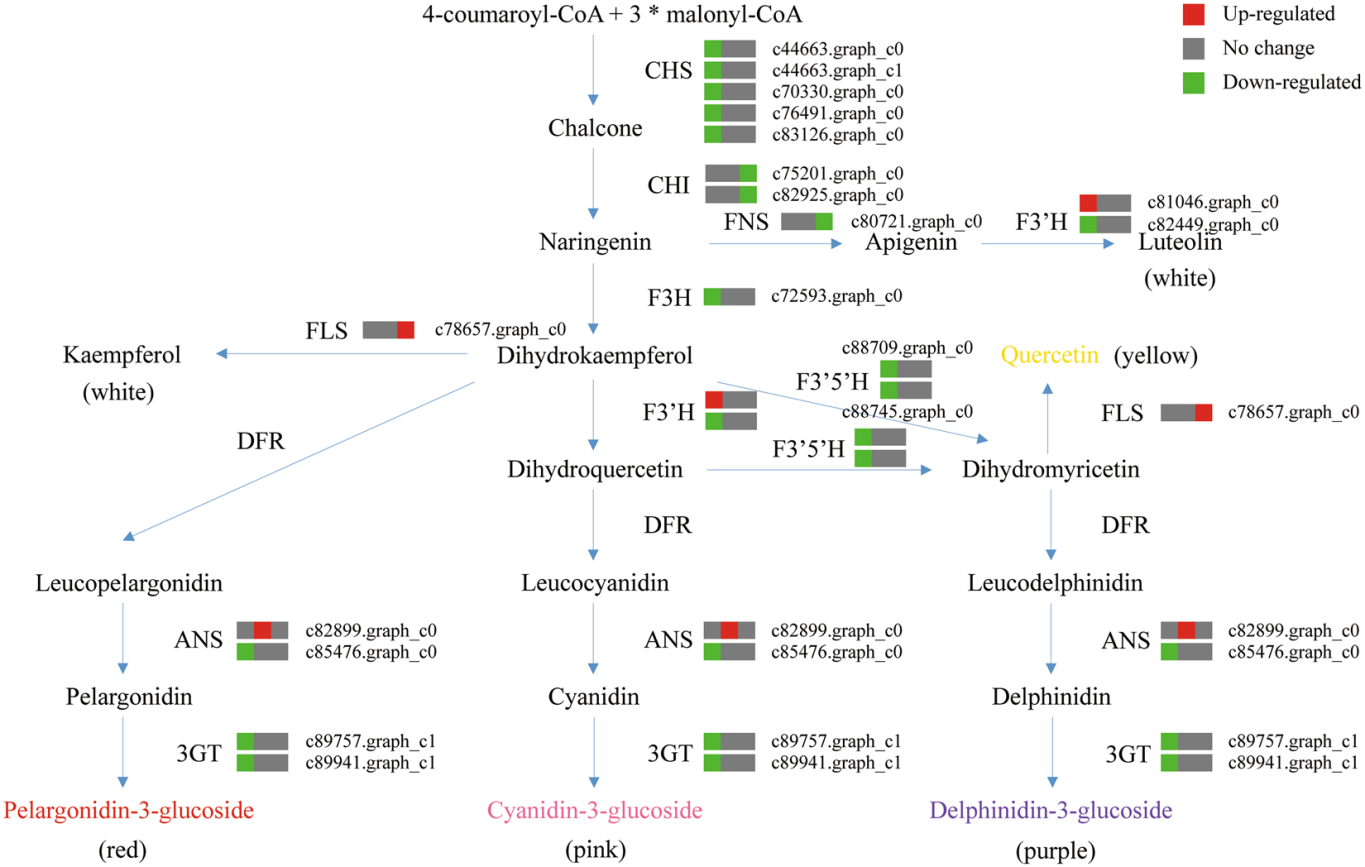
The enzyme-coding structural genes involved in the main pathway of flavonoid biosynthesis in *C. morifolium* ‘Chuju’. A total of 18 unigenes with the annotation of nine structural genes were differentially expressed during flower development, where red indicates up-regulated, green indicates down-regulated and gray indicates no change. The three squares stand for the early, middle and mature growth phases in turn. CHS: chalcone synthase, CHI: chalcone isomerase, FNS: flavone synthase, F3H: flavanone 3-hydroxylase, F3′H: flavonoid 3′-hydroxylase, F3′5′H: flavonoid-3′, 5′-hydroxylase, FLS: flavonol synthase, DFR: dihydroflavonol 4-reductase, ANS: anthocyanidin synthase, and 3GT: UDP-glucose-flavonoid 3-O-glucosyltransferase.

Based on the above analysis of DEG profiling, a majority of the structural genes were expressed throughout flower development. Besides, there are two unigenes (unigene ID: c101558.graph_c0, a member of *CHS* homologies; unigene ID: c33294.graph_c0, a member of *FLS* homologies) that were not always expressed (Fig. 7). Among them, 18 homologous members were identified as DEUs and their expression patterns were compared in detail (Fig. 8). Interestingly, differential expression of all these unigenes was detected only once among the three comparisons and a majority of them were significantly down-regulated in the early growth phase.

**Figure 9:**
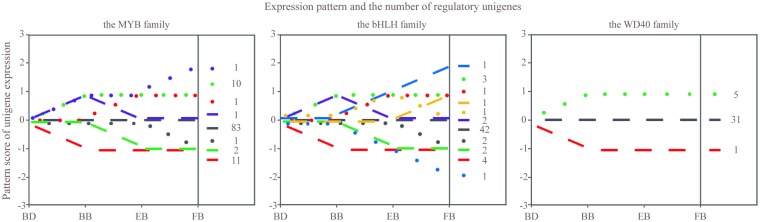
Expression pattern and the number of regulatory unigenes involved in the flavonoid biosynthetic pathway during flower development of *C. morifolium* ‘Chuju’, including the budding (BD), bud breaking (BB), early blooming (EB) and full blooming (FB) stages. While a given unigene is up-regulated, the pattern score will plus one. While a given unigene is down-regulated, the pattern score will minus one. While a given unigene has no change in expression, the pattern score will keep invariant.

Using the pattern score described by Muhlemann^25^, we also found that *MYB*, *bHLH* and *WD40* were expressed in 8, 11 and 3 different patterns, respectively (Fig. 9). Overall, the expression of most unigenes did not significantly fluctuate throughout flower development as they fell into the pattern score {0,0,0}. While with regard to the DEUs, a majority fell into the patterns {1,0,0} and {−1,0,0}, indicating that these unigenes were differentially expressed from BD to BB stages. Interestingly, the expression of *WD40* only fell into the patterns {1,0,0} and {−1,0,0}.

### Quantitative real time PCR validation of gene expression

**Figure 10:**
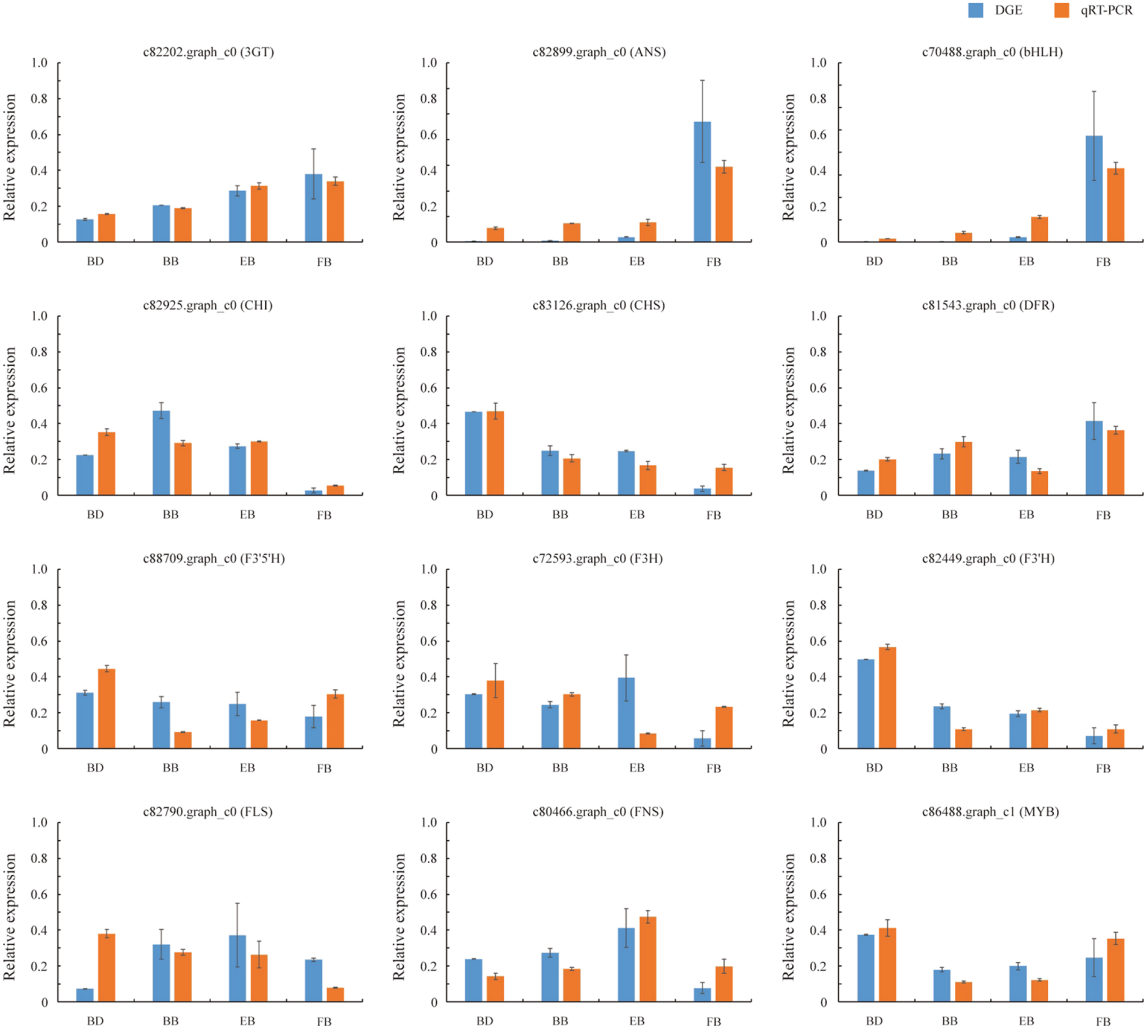
The expression levels and patterns of 12 randomly selected unigenes were confirmed by qRT-PCR during flower development of *C. morifolium* ‘Chuju’.

To confirm the high-throughput sequencing data, quantitative real-time PCR (qRT-PCR) was employed as an effective method due to its sensitivity, specificity and rapidity^26^. Specifically, 12 unigenes putatively participating in the flavonoid pathway were randomly chosen for validation of their expression levels and patterns at different developmental stages. As a result, the qRT-PCR analysis revealed that most of the tested unigenes had a strong correlation with the expression data obtained from RNA-Seq assay (Fig. 10). By contrast, two unigenes (c72593.graph_c0 and c82790.graph_c0) showed inconsistent patterns between qRT-PCR and RNA-Seq data. The reason might be that these two genes are expressed at extremely low levels at certain stages, which tends to bring error rates^27^. Specially, the contradiction is mainly from the EB stage in c72593.graph_c0 gene expression and the BD stage in c82790.graph_c0 gene expression.

## Discussion

For the transcriptome of mixed samples from four sequentially developmental stages of *C. morifolium* ‘Chuju’ flowers, approximately 7.77 Gb of high-quality data were obtained and assembled into 63,854 unigenes with an average length of 741 bp. Among them, 34,605 unigenes were assigned a specific or general function on the basis of comparison against sequences in the nr, Swiss-Prot, KEGG, COG and GO databases, corresponding to approximately 54.19% of the total unigenes. This value is similar to that in *C. morifolium* ‘Fenditan’ (47%)^16^, *C. morifolium* ‘Yuuka’ (60%)^17^ and *C. lavandulifolium* (53%)^28^ based on an equivalent strategy of transcriptome analysis.

Subsequently, DGE profiling was performed in the individually developmental stages. Due to a lack of genomic information of *C. morifolium*, the sequencing data were aligned against the unigenes assembled from previous transcriptome analysis. As mentioned above, flowers from the BD, BB, EB and FB stages shared 42,348 unigenes in common, indicating that the majority of unigenes were constantly expressed across the entire developmental stages. Meanwhile, comparative analysis further demonstrated that the expression levels of most unigenes were generally stably throughout flower development. Undoubtedly, the constant and stable expression of unigenes should suggest their essential roles for maintaining flower development. By contrast, differentially expressed unigenes might imply their regulatory function during flower development in *C. morifolium*.

Actually, flavonoid biosynthetic pathway was well established in model species. CHS is the first committed enzyme specific for the flavonoid biosynthesis^29^. Then, the following steps catalyzed by CHI, FNS, F3H, F3′H, F3′5′H, FLS, DFR, ANS and 3GT will lead to production of different flavonoid subgroups by modifying molecular skeleton and/or backbone. In this work, homologous unigenes of fourteen *CHS*, four *CHI*, two *FNS*, ten *F3H*, seven *F3*′′*H*, eighteen *F3*′*5*′*H*, seventeen *FLS*, eight *DFR*, four *ANS*, and thirteen *3GT* were identified (Table 3). The average values of their expression levels are 33.3, 31.4, 27.9 and 11.6 in the four successive stages of flower development, respectively. Using the Wilcoxon test, we found that these values except for 11.6 are significantly higher than the average values of 14.5, 13.1, 13.3 and 13.0 for the entire unigene expression levels in each developmental stage (*P*-value < 1*e*-10). It should be noteworthy that a higher expression level of a gene stands for a more important function in a given cell^30^. Meanwhile, different members from the same families of structural genes might be responsible for enzymes that differ for substrate specificities^31^. Therefore, these results could partially explain why a variety of flavonoid compounds were accumulated in the dry *C. morifolium* ‘Chuju’, and might indicate that the biosynthesis of flavonoids is enhanced at the beginning but not in the process of flowering.

Furthermore, we observed that the ray florets undergo a gradual change in color, ranging from yellow to white during the flower development (Fig. 1). This change is due to an increase in flavone and flavonol content as well as a possible decrease in anthocyanin accumulation^32^. Chemical component analysis also revealed that the dry capitulum is rich in flavone and flavonol, such as acacetin, apigenin, luteolin and quercetin^33^. Hence, it is believed that there may be a dynamic variation of dominate metabolic pathway at different stages of flower development in *C. morifolium* ‘Chuju’. Coincident with the reduction of anthocyanin content, a majority of structural genes involved in the flavonoid biosynthesis were significantly down-regulated in the early growth phase as shown in Fig. 8. On the other hand, the only up-regulated gene *FLS* (unigene ID: c78657.graph_c0) in the mature growth phase, will make the flowers produce more amount of flavonols. These findings indicated that flavonoid biosynthesis might be synchronously controlled by the process of flower development.

Generally, the relationship of branched metabolic pathways involved in flavonoid biosynthesis could be regulated by the common upstream signaling. Emerging evidence has shown that the expression of structural genes participating in the flavonoid biosynthetic pathway is tightly regulated during flower development. To date, the ternary MYB-bHLH-WD40 (MBW) transcriptional activator responsible for the regulation of flavonoid metabolic pathway is well characterized and highly conserved throughout the plant kingdom. In Arabidopsis, TT2 (a MYB transcription factor), TT8 (a bHLH transcription factor) and TTG1 (a WD40 repeat protein) could interact with each other to form a MBW complex and subsequently activate the expression of downstream genes involved in flavonoid metabolism to promote the biosynthesis of proanthocyanidin in the seed coat^34^. In grape, VvMYBA1 and VvMYBA2 could promote anthocyanin biosynthesis by activating the expression of an anthocyanin acyltransferase^35^. In apple, MdbHLH3 could also increase anthocyanin biosynthesis through activating the expression of structural genes in isolation^36^. Nonetheless, some members of the MYB family, such as MYBL2 in Arabidopsis^37^, VvMYBC2 in grape^38^, MYB182 in poplar^39^, MYB27 in petunia^40^, GmMYB100 in soybean^41^, may act as repressors to negatively regulate the biosynthesis of flavonoids by interfering with the formation of functional MBW complex.

In the present study, a number of unigenes homologous to *MYB*, *bHLH* and *WD40* were identified with diverse expression patterns. While the different expression patterns of unigenes usually stand for their different roles in regulatory network^30^, the huge expression variation of regulatory unigenes in the early growth phase might largely attributed to the metabolic adjustments at flower bud developmental stage (Fig. 9). Notably, these findings are in line with previous expression analysis that a majority of the structural unigenes were significantly regulated and the metabolic flux was redistributed towards a production of more flavonols following the flower bud developmental stage. Therefore, an implication is that in *C. morifolium* ‘Chuju’, like in many other plant species, the ternary MBW complex may also play important roles in controlling the biosynthesis and composition of flavonoids along with flower development.

**Figure 11:**
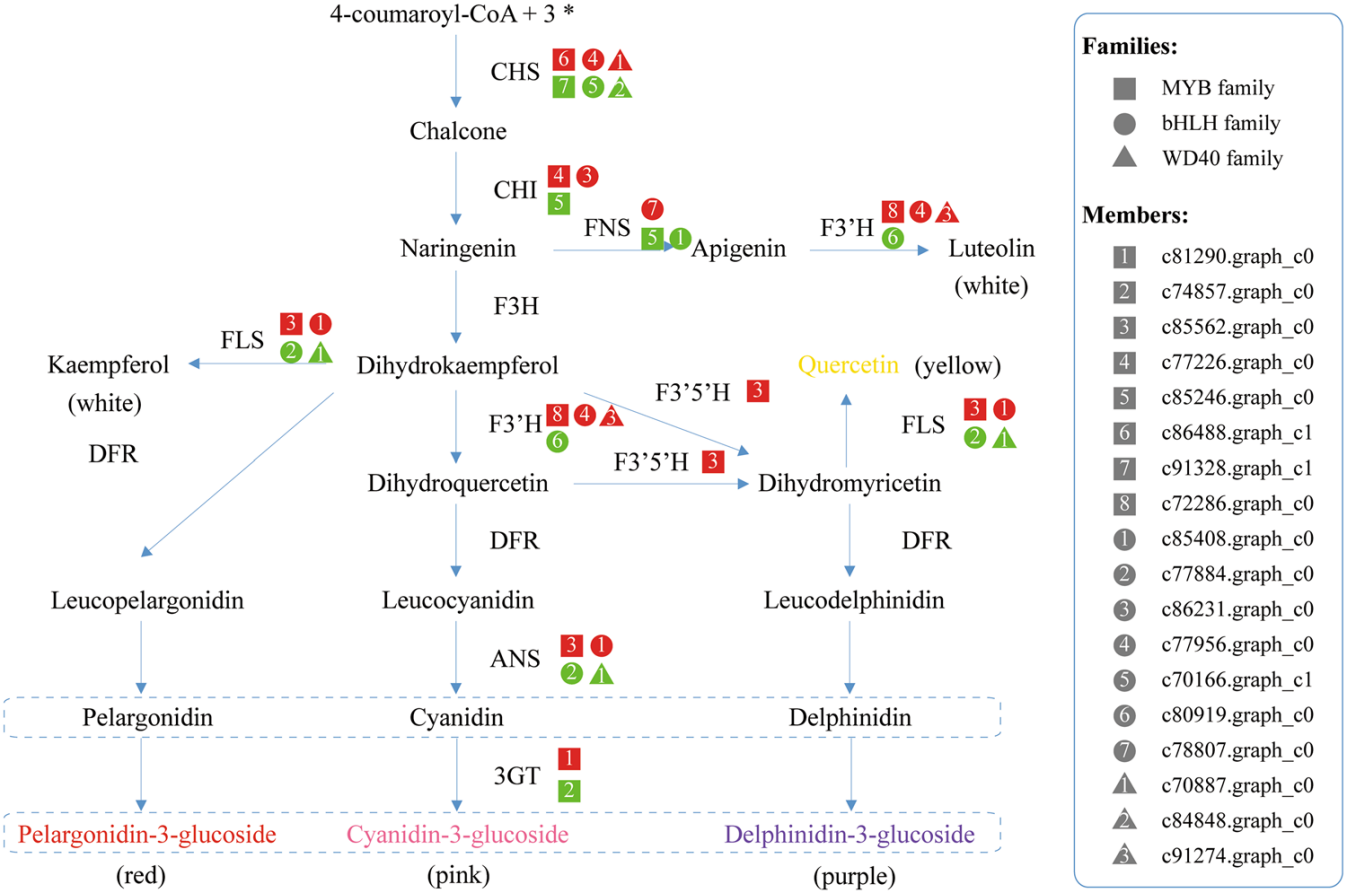
The potential regulatory relationships between MBW complex and structural genes. The square denotes MYB family, the circle denotes bHLH family, and the triangle denotes WD40 family. Numbers in the middle stand for different members in each protein family. Red indicates positive regulation and green indicates negative regulation. CHS: chalcone synthase, CHI: chalcone isomerase, FNS: flavone synthase, F3H: flavanone 3-hydroxylase, F3′H: flavonoid 3′-hydroxylase, F3′5′H: flavonoid-3′, 5′-hydroxylase, FLS: flavonol synthase, DFR: dihydroflavonol 4-reductase, ANS: anthocyanidin synthase, and 3GT: UDP-glucose-flavonoid 3-O-glucosyltransferase.

To further explore the functional relationships between MBW complex and structural genes, we performed a gene co-expression network analysis and built a potential regulatory network. Each of the structural genes with any available regulatory genes were defined as a “network motif”. As a result, 15 network motifs including 18 regulatory genes and 8 structural genes were constructed (*P*-value < 0.01) (Fig. 11; Supplementary Table S9). Our data support that different members of the MBW complex might undergo different regulatory mechanisms with either positive control or negative control. Also, different members or combinations are likely responsible for different enzymes. For example, *CHS* might be regulated by MBW complex and *3GT* could be regulated by the MYB transcriptional factor independently. Specially, the common function of WD40 proteins is coordinating multi-protein complex assemblies. As shown here, no WD40 proteins were identified alone, but always associated with the MYB or bHLH transcriptional factors.

In summary, the comparative transcriptome analysis of *C. morifolium* ‘Chuju’ has revealed that the biosynthesis of flavonoids is detected from the beginning of flower bud formation and tightly regulated by the MBW complex throughout diverse flower developmental stages. After flower bud development, the distribution of metabolic flux was varied towards different branches in the flavonoid biosynthetic pathway. Many regulatory and structural unigenes possibly participating in flavonoid biosynthesis were identified and bridged, which could provide valuable genetic resources for studies of metabolic pathway, nutrition improvement and transgenic breeding in chrysanthemum.

## Methods

### Plant material and sample collection

*C. morifolium* Ramat. cv. ‘Chuju’ was grown under natural conditions in the experimental field of Chuzhou University at Chuzhou, Anhui province, People’s Republic of China (N32°17′, E118°18′). The annual average of temperature and precipitation is approximately 15.42 °C and 1,054 millimeters, respectively. Based on the pre-existing study^42^, flower cuttings were collected from four sequentially developmental stages: budding (BD), bud breaking (BB), early blooming (EB) and full blooming (FB) stages (Fig. 1). The BD stage started on around Oct 25th and the subsequent stages are approximately one week later in turn. Meanwhile, multiple flowers from three plants were sampled for biological replicates. After harvest, these flower tissues were frozen immediately in liquid nitrogen and stored at −80 °C until further required. The location of this field nursery is not privately-owned or protected in any way.

### RNA extraction and high-throughput sequencing

Total RNAs were extracted from flower tissues with the TRIzol reagent (Invitrogen, MA, the United States) by following the manufacturer’s protocol. RNA quality and quantity were evaluated using an Agilent 2100 Bioanalyzer device (Agilent, CA, the United States). Only the samples with a λ_260/280_ ratio of 1.8–2.1 and a λ_260/230_ ratio of 2.0–2.5 were retained. Next, RNase-free DNase I (Takara, Dalian, China) was used to remove the contaminating DNA, and oligo (dT) coated magnetic beads was mixed to separate the poly (A) fraction. The obtained mRNA molecules were sequentially broken into short fragments and converted to cDNA by DNA polymerase I (Takara, Dalian, China) through the reverse transcription polymerase chain reaction (RT-PCR) amplifications. The reaction mixture was subjected to polyacrylamide gel electrophoresis (PAGE) and the suitable fragments were isolated and purified. Finally, 30 µg library that has been equivalently pooled from the four stages of flower tissues was submitted for whole transcriptome sequencing and about 10 µg libraries remaining from each flower tissue were directly used for digital gene expression sequencing. All the high-throughput sequencing assay was performed using the HiSeq. 2000 system (Illumina, CA, the United States) at the Biomarker Institute (Beijing, China) with two independent technical replicates.

### Transcriptome assembly and gene annotation

After removing the 5*′* adaptor, trimming the 3*′* acceptor, filtering those low quality reads with a quality value (Q) less than 10 (the quality value was calculated as following: Q = ASCII character code – 64) and cleaning up contaminated reads^43^, we consequently obtained the clean reads. First, the distinct contigs were assembled with the short reads by using the software program SOAP2^44^. Then, these reads were aligned back to the assembled contigs, which could detect those contigs from the same transcript. Next, scaffolds between closed contigs were constructed by employing the paired-end mapping analysis. Finally, paired-end reads were used again to fill the intra-scaffold gaps and a set of non-redundant unigenes were constructed with the least amount of unaligned reads.

For functional annotation, the BLAST program (*E*-value = 1*e*−5) was conducted between unigenes and various nucleotide/protein databases, namely the nr, Swiss-Prot, KEGG, COG and GO databases. Within the alignment against each database, the best aligning results were reserved. Specifically, when results from different databases conflicted with each other, a priority order of nr, Swiss-Prot, KEGG, COG and GO was followed to determine the annotated unigenes. Subsequently, the Blast2GO pipeline^18^ and WEGO online tool^45^ were performed to assign and compare GO terms of unigenes in turn. Furthermore, the transcription factors were predicted by the iTAK online program^22^.

### DEU identification and enrichment analysis

To eliminate any possible bias generated from variation in sequence length, the expression levels of unigenes were calculated using the RPKM method^46^. DEUs were identified between every two successive stages based on a DESeq algorithm^47^. The false discovery rate (FDR) method^48^ was applied to correct for *P*-value when calculating the differential expression in multiple tests. Obviously, the smaller FDR and higher ratio of a given unigenes represent the larger variation in expression. Here, an FDR threshold of <0.01 and a fold-change threshold of >2 were selected to define the significant differences in unigene expression.

Subsequently, the identified DEUs were assigned to GO functional annotation. The enrichment analysis of each GO term was performed using the Hypergenometric test as follows:

$${f}({k}\,;\,{n},\,{m},\,{N})=\frac{(\begin{array}{c}{m}\\ {k}\end{array})(\begin{array}{c}{N}-{m}\\ {n}-{k}\end{array})}{(\begin{array}{c}{N}\\ {n}\end{array})}$$

$$P \mbox{-} value=1-{f}({k}\,{;}\,n{,}\,m{,}\,{N})$$

where *N* was defined as total number of annotated GO terms in all the unigenes, *M* was total number of a specific GO term in all the unigenes, *n* was total number of GO terms in all the DEUs, *k* was total number of a specific GO term in all the DEUs.

### Building a gene regulatory network

The potential gene regulatory network was constructed based on the assessment of co-expression intensity between regulatory genes and structural genes as described in our previous work^49^. For each assessment, all the DGE data across the four sequentially developmental stages were extracted and used. Co-expression intensity was calculated as a Pearson’s correlation coefficient (*R*), which takes a range of values from +1 to −1. In the current study, an *R*-value of 0.8 and *P*-value of 0.01 were firstly employed as thresholds. Then, positive maximum values and negative maximum values were retained, which stand for the most likely positive regulation and negative regulation, respectively.

### Verification by qRT-PCR analysis

The expression patterns of unigenes in the RNA-Seq analysis were further confirmed by qRT-PCR method using an Applied Biosystems StepOne^TM^ Real-Time PCR System (Applied Biosystems, MA, the United States) and a FastStart Universal SYBR Green Master (Roche, Basel, Switzerland). A total of 12 unigenes that participate in the flavonoid biosynthetic pathway were randomly chosen and their appropriate primers were designed by using the Primer5 software (Table 4). The reactions were performed in a 96-well optical plate using the following conditions: an initial polymerase activation step for 30 s at 95 °C, 40 cycles of 5 s at 95 °C for denaturation, 20 s at 60 °C for annealing and elongation. After reaction, the threshold cycle (Ct), defined as the fractional cycle number at which the fluorescence passed a fixed threshold, was determined using the default threshold settings. Relative expression levels were calculated by the ${2}^{-{\rm{\Delta }}{\rm{\Delta }}{C}_{{\rm{T}}}}$ method^50^. Each sample of the BD, BB, EB and FB tissues that were originally used for RNA-Seq assay was represented with three biological replicates. The *catalytic subunit of protein phosphatase 2A* (*PP2Acs*) encoding gene (unigene ID: c85200.graph_c0) was used as an internal reference (Table 4)^51^.

**Table 4:** Primers used for the qRT-PCR analysis.

Unigene ID	Forward primer (5*′* - 3*′*)	Reverse primer (3*′* -5*′*)
c82202.graph_c0	CGCAAGCCACCACCTAAAGAC	GTGCCCAACCGCAAACCA
c82899.graph_c0	AAAAGGGTCTGGCGGGAGTTC	ACCCCAATACGGCTGCCATAA
c70488.graph_c0	GCATTGCTACCTGCTTCAAGTTCTA	CCCATCGTTTTCCATAGAATCCA
c82925.graph_c0	ATGGGTTCAGAAATGGTTATGGTTG	GCTCGGTTCCAGATTTACCCTTC
c83126.graph_c0	TCAAGGAGGAGAAGATGAGAGCC	CCAGGACCGAACCCGAATAA
c81543.graph_c0	ATTAGCATTTGAGAACCCCGAAG	CAAGAGATGGGTAGAGTTCGGCT
c88709.graph_c0	TCAACCTTATTGGCACCCTTCC	GCGACAAGAACATTAAACGACCC
c72593.graph_c0	TCAAGCGACTCGTGATGGTG	AATCTTCCGTTGCTCAAATAATGT
c82449.graph_c0	ATGGGCAATAGCCGAACTCA	GGAGCCTAAACACCTCTTTCACA
c82790.graph_c0	GAATTAAGGGAGGCATGTGAAGAA	AACACCAAGGGCTAAGTCAGGAG
c80466.graph_c0	CTTATCCACCAGTCCTTTCACAGTC	AAGGCGACGCCATAAGTTACAT
c86488.graph_c1	GCAAGTCATAACCCGAAACGAG	CTGATTACACCACCTTAACCTACACG
c85200.graph_c0	ATTTCTTCCTCTTGCGTGGTAAC	GGTCGGGTGACAATCCTCC

## Electronic supplementary material

Supplementary Table
